# Evaluation of the impact of leadership development on nurses and midwives underpinned by transformational learning theory: a corpus-informed analysis

**DOI:** 10.1108/LHS-09-2022-0092

**Published:** 2023-06-30

**Authors:** Carmel Bond, Gemma Stacey, Greta Westwood, Louisa Long

**Affiliations:** Research and Policy Unit, Florence Nightingale Foundation, London, UK and Nursing and Midwifery, College of Health Wellbeing and Life Sciences, Sheffield Hallam University, Sheffield, UK; Research and Policy Unit, Florence Nightingale Foundation, London, UK; Florence Nightingale Foundation, London, UK; Research and Policy Unit, Florence Nightingale Foundation, London, UK

**Keywords:** Leadership development, Nurses, Midwives, Allied health-care professionals, Transformational learning theory, Health-care systems

## Abstract

**Purpose:**

The purpose of this paper is to evaluate the impact of leadership development programmes, underpinned by Transformational Learning Theory (TLT).

**Design/methodology/approach:**

A corpus-informed analysis was conducted using survey data from 690 participants. Data were collected from participants’ responses to the question “please tell us about the impact of your overall experience”, which culminated in a combined corpus of 75,053 words.

**Findings:**

Findings identified patterns of language clustered around the following frequently used word types, namely, confidence; influence; self-awareness; insight; and impact.

**Research limitations/implications:**

This in-depth qualitative evaluation of participants’ feedback has provided insight into how TLT can be applied to develop future health-care leaders. The extent to which learning has had a transformational impact at the individual level, in relation to their perceived ability to influence, holds promise for the wider impact of this group in relation to policy, practice and the promotion of clinical excellence in the future. However, the latter can only be ascertained by undertaking further realist evaluation and longitudinal study to understand the mechanisms by which transformational learning occurs and is successfully translated to influence in practice.

**Originality/value:**

Previous research has expounded traditional leadership theories to guide the practice of health-care leadership development. The paper goes some way to demonstrate the impact of using the principles of TLT within health-care leadership development programmes. The approach taken by The Florence Nightingale Foundation has the potential to generate confident leaders who may be instrumental in creating positive changes across various clinical environments.

## Introduction

At a time of global health challenges such as the nursing and midwifery staffing shortage and as we recover from the COVID-19 pandemic, there has never been a greater need for effective leadership across all tiers of the health and social care workforce (Abbas, 2021; Joseph-Richard and McCray, 2022; Messenger, 2021; Richard *et al.*, 2021). Yet, despite a growing need for leadership development in nursing and midwifery, previous studies have argued there is limited scholarship in relation to how leadership capability might be achieved across wide-ranging and diverse contexts (Miles and Scott, 2019).

The Healthcare Leadership Model (HLM) (Boyatzis, 1982) has provided a basis for the UK’s national strategy for leadership development (NHS England and Health Education England, 2018). HLM describes what leaders “do”, including inspiring others; inspiring a shared purpose; and development of “the self” as role model who can naturally influence others to adopt a shared vision for health-care quality improvement. Arguably, the context of nursing and midwifery leadership can be conceived as a social process in which individuals are considered social actors, engrained within interwoven social realities from which meaning is made and from which they base their perceptions and actions (Iwowo, 2016; Oates, 2012). Hence, it is important that, to develop as leaders, nurses and midwives gain insight into their existing values and personal qualities, regarding how the “self” might be experienced by other individuals with whom they work (Gilmartin and D’Aunno, 2007). This can be achieved through programmes which enable individuals to develop both professionally and personally, whilst building increased self-awareness (*see* Bond *et al.*, 2022) and self-efficacy.

With regard to leadership theory, previous programmes have been established based on research that has expounded traditional leadership theories, for example, Transformational Leadership Theory [1] (Burns, 1978) is noted to have held prominence across the health-care leadership literature for the past few decades (Paton *et al.*, 2021; Wong *et al.*, 2013), specifically in relation to nursing leadership (Crowne *et al.*, 2017). The programmes we report on here are underpinned by the principles of Transformative Learning Theory (Mezirow, 2000), which focuses on a process of continual critical reflection, through which there is a “dramatic, fundamental change in the way we see ourselves and the world in which we live” (Cranton, 2016, p. 318). Hence, it was anticipated that individuals embarking on one of The Florence Nightingale Foundation’s programmes would be changed in profound and long-lasting ways through a process where they were encouraged to challenge existing and habitual psychological, sociocultural, and epistemic meaning perspectives about “the self”, as well as exploring the individual differences in meaning perspectives of each other.

In the current study, we amalgamated feedback (in the form qualitative comments) from 690 participants from across a collection of 13 of “The Florence Nightingale Foundation’s(FNF)” leadership development programmes. The aim was to evaluate the impact of FNF’s approach to developing health-care leaders.

### Overview of The Florence Nightingale Foundation’s programmes

FNF’s programmes aim to enable nurses and midwives to develop personal confidence in their expertise and authority as health-care leaders. Each programme aims to equip them with the skills to generate and use evidence and articulate their knowledge in a manner that impacts policy, practice and to promote clinical excellence in their chosen field of practice. This is fulfilled through a comprehensive programme using a variety of experiential leadership development activities. Programmes are tailored to the experience and area of practice the nurses and midwives are working within. However, all programmes share the following learning objectives:

- Demonstrate enhanced awareness of how personality preferences influence personal effectiveness and performance in teams.
- Identify and critically appraise opportunities to influence through personal and collective authority.
- Develop strategies to express self in a manner which communicates presence, enables influence, and has impact.
- Explore personal courage and develop tools for staying effective under pressure.
- Formulate and put into practice plans which contribute to improved outcomes for patients and staff underpinned by evidence and quality improvement methodology.

Programmes are typically 6 months in duration and throughout each programme, participants are encouraged to better understand “the self” (and others) through individual and group work where they engage in a process of introspection and critique of “the self”.

FNF is a learning organisation, and, as such, it captures participants’ feedback through questionnaires which are collected prior and after the delivery of leadership programme(s). Information gathered from stakeholders and participants is used to inform further improvements and demonstrate the impact of the service offered by “FNF”. Based on information gathered through pre-programme collaboration with providers and commissioners, course topics are carefully selected to meet the evolving learning needs of health-care organisations such that “FNF” can design and deliver programmes that address the most significant issues impacting on the progression of the nursing and midwifery professions. By evaluating the experiences and perspectives of individuals who have undertaken these programmes this paper will provide insight into the usefulness of employing transformational “learning” theory as an approach to developing future health-care leaders.

### Ethical considerations

As part of the process of consenting to data administration, FNF’s programme participants are informed that their names, contact details will be collected and that their de-identified feedback may be used in evaluation and for the improvement of future leadership programmes, as per the privacy policy and as a condition of their sponsorship. As such, implicit informed consent was obtained from all programme participants and no formal ethics committee approval was sought; only anonymised feedback data was collected. Once data was collected via FNF’s website, it was stored on a secure cloud server which is only accessible to FNF’s employees in accordance with European Union General Data Protection Regulations (UK Government, 2018).

### Data collection

A total of 1,240 participants joined FNF’s leaderships programmes between 2019 and 2021. Online surveys were used to capture participants’ feedback during the programme and immediately following completion of the programme. All participants were provided with the same survey, which comprised questions about demographics; job role; Agenda for Change [2] banding; self-identified learning objectives; and self-reported scores in relation to individually perceived self-efficacy (Schwarzer and Jerusalem, 1995). Participants were also provided with space to provide qualitative feedback comments in response to the question: “Please tell us about the impact of your overall experience”. Questionnaire data were organised into separate Excel workbooks and categorised into the individual leadership programmes. Qualitative data were then extracted from each participant’s individual response and transferred to a single Microsoft Word document (corpus).

### Data analysis

Due to the size of the corpus (75,053 words), some computational means were required to draw out key topics, frequently referred to within the qualitative data, and gain insight into the context of the comments. Natural Language Processing is a field in Computer Science which uses models and machine learning to make sense of large volumes of unstructured free text. The latter sought to determine the most frequently used words and their sentiment using corpus linguistics (Selvi, 2019) to identify topics and analyse themes. This study used AntConc 3.5.9 (Anthony, 2023) a freely accessible online corpus analysis toolkit for processing language. This toolkit can be used to scan a large volume of words to detect word and phrase patterns within them, and automatically cluster the words into groups and identify any collocated words.

## Results

Data in the form of naturally occurring language is valuable for obtaining contextual insight into participants’ experiences and impact of “FNF’s” programme (Selvi, 2019). This free text data was analysed with the view that an analysis of language offers a way of evaluating participants’ experiences as well as providing insight into how participants reflected upon themselves as leaders (during the process of learning and reflecting upon the developing “self”). Using AntConc 3.5.9 (Anthony, 2023) software, a collocation analysis was computed, which resulted in the following notable word types that were frequently located across the corpus: confidence; influence; self-awareness; insight; and impact.

## Collocation analysis

The words “confidence” and “confident” were noted a total of 213 times across the corpus to be collocated with the words “developed”, “increased”, “built”, “enabled”, “boosted”, “grown”, “aided”, “provided” and “gained”. There was also a notable pattern of language in relation to the word “leadership” (and lexical lemmas of leadership, i.e. “leading”, “lead”, “leader”), which was collocated with the words “confidence” and “confident”. The latter collocation(s) was observed a total of 29 times and outlines the way in which participants constructed their identity, as leaders, in relation to their overall leadership experience of the The Florence Nightingale’s programme. Increased confidence was remarked upon in relation to feeling more “effective” in their existing role, as well as improving communication skills, motivation and individual agency within their role. These results illustrate the influence of FNF’s leadership programme in relation to the way in which participants viewed “the self” after having completed and/or as they emerged from the programme. A distinct transformation of “self” in participants’ use of language, in relation to themselves, is remarkable, specifically in relation to the growth and development of individual confidence as leader. This provides evidence to suggests that the FNF’s programmes are effective in developing confident future health-care leaders (Table 1).

The potential for wider impact, in relation to patient care was observed within participants’ discourse, e.g. “It has also increased my confidence in speaking out and making changes to patient care” and “This course has given me the confidence of providing the best care for patients”. FNF’s underpinning rationale for using transformational learning theory (TLT) (Mezirow, 2000) as an educational approach to developing health-care leaders was the idea that reflection on the “self” might permeate more broadly, after having completed the programme, in terms of participants’ perceived capability to view themselves as change agents. Along these lines, the key word “influence” was noted 43 times in the corpus. A total of 15 concordance lines illustrated participants’ reflections both in terms the externalisation of “self-forming” activity and personal beliefs about their capacity for wider impact (through the use of phrases such as, “shape my”, “transformed my perspective”, “made me”, “am able”, “I can” and “able to”) (Table 2).

Linguistic expression in relation to the “self” was also notable in a third pattern of language use, in which participants were observed to have referred to the development of awareness of “the self” as leader. A total of 28 concordance lines were retrieved from the corpus where the key word “awareness” was observed. A total of 15 of these concerned heightened self-awareness as a result of the FNF’s programme (Table 3).

The word “insight” was used 29 times, and in relation to what participants had achieved from taking part in the programme. This was collocated around the following lexical lemmas, “gained”, “gain”, “gave” “given”, “get” 12 times, which demonstrated that participants believed they had received insight into various aspects of leadership in health care. This understanding included thinking about strategic elements of health-care environments, whilst also gaining a deeper understanding of the self as future leader (Table 4).

Two final patterns of language, notable across the corpus was formed around the word “impact”. This was observed to occur a total of 119. Participants’ evaluations on their overall experience, after having undertaken the (FNF leadership) programme, clearly illustrated a profound sense of learning, and continued questioning, in relation to the self and impact of the self on others (Table 5).

The word “impact” was closely collocated with the word “positive”, in relation to the development of the self as “leader”, and in the sense of the individual potential for wider impact as a result of the (FNF’s) programmes (Table 6).

This perceived outcome suggests participants recognised how the confidence, awareness and insights they have gained as a result of the programme goes beyond individual and relational impact and extends to the practice environment where they are impacting on patient care.

## Discussion

In an effort to assess the overall impact of FNF’s programmes for developing health-care leaders, and the feasibility of this approach (using TLT), the current study examined survey data from 690 participants who undertook one of FNF’s programmes in the past two years. As far as we know, this is the first paper to report on programmes designed to develop health-care leaders, where transformational “learning” has been used, using corpus linguists as a research method (Mezirow, 2000). Kirkpatrick’s (1959) evaluation model has been used previously to measure the impact of the leadership development programmes (*see* Alsalamah and Callinan, 2021). However, the method applied here goes beyond reaction to demonstrate explicit accounts of learning, and descriptions of behaviour changes that are both unique to individuals and observable across participants’ discourse.

Our findings suggest that those who participate in FNF’s programmes experience growth in their perceived leadership capabilities and competencies. In relation to the way in which participants perceived a sense of themselves as leaders, the collocation analysis identified word types that were closely related to an increasing understanding of the of self as efficacious, e.g. confident, influential, self-aware, insightful, and considering “the-self” as able to have impact on others, their practice environment and ultimately patient care. This language is reflective of the components of self-efficacy which is defined as “an individual's belief in their ability to successfully perform a task or achieve a desired outcome” (Bandura, 1997). Bandura’s four essential concepts in self-efficacy are mastery of experiences, social modelling, social persuasion and psychological responses (Bandura, 1977, 1986, 1997). Mastery of experiences is linked to description of impact in practice, social modelling to self-awareness, social persuasion to perceived influence and psychological responses to perceived confidence.

Increased self-efficacy has been shown to be a powerful predictor of leadership performance; nurses’ belief in individual ability to influence professional practice; self-confidence in their own decision(s) relating to the professional practice environment (*see* Moran *et al.*, 2021). Hence, our findings highlight the need for leaders to develop self-efficacy in the context of health-care delivery. Any future research relating to this particular programme must therefore be able to demonstrate a clear change in participants’ self-efficacy, e.g. pre and post programme ratings accurately matched to individual participants.

The collocation analysis indicated that participants’ self-forming perceptions of themselves as leaders had the potential for much wider impact of patient care and within the wider clinical context. However, without conducting further realist evaluation and longitudinal research, this is impossible to ascertain from this study. A realist approach would offer insight into both the individual and organisational mechanisms of change, as outlined by De Brún and McAuliffe (2022), and account for the complexity of diverse health-care environments. Nevertheless, the word-types used by participants in the current study, for example, the final two patterns of language use observed participants’ expressing a high level of reflection, indicated “theoretically” the notion of transformation of the self in terms of individuals beginning to consider critically their own worldview and how this might impact leadership (Cranton, 2016). However, this must be interpreted with caution as it is unclear whether this transformation can be sustained over time, for example, when the participants had completed the programme and returned to clinical work. Furthermore, it is difficult to ascertain to what extent prevailing cultural values might also determine how leadership identity is developed and maintained over time (Joseph-Richard and McCray, 2022).

Regardless, participants clearly experienced growth in terms of increased confidence and a sense of having changed as a result of the programme. However, it would be beneficial to design future surveys using measures that can assess changes in self-confidence with greater accuracy, which may offer more credence to the results in terms of validating and triangulating the data presented here. An area, which The Florence Nightingale Foundation has already improved upon, and which will help future evaluations, is the inclusion of additional demographic information such as age, gender and ethnicity. This will enable more sophisticated analysis of the possible relationship between these factors and indicators such as satisfaction ratings, attainment of learning goals and job promotion and or career progression. Demographics may also facilitate targeted improvements in future programme design, content and delivery for specific groups.

Ultimately, this paper has captured the benefits of The Florence Nightingale Foundation’s leaderships development programme. The insights gained from the collocation analysis illustrate the socially constructed nature of the concept of health-care leadership. This analysis has clearly highlighted the benefits of applying TLT (Mezirow, 2000) is an approach to developing highly effective future health-care leaders.

## Strengths and limitations

The use of corpus assisted technology to analyse large amounts of qualitative data is easy to repeat and verify. This approach also mitigates “human” biases is the analysis of the textual comments from survey respondents. Although, human interaction and human interpretation with the data could be viewed as potentially introducing bias to the analytical process (Piepenbrink and Gaur, 2017). The size of the corpus (75,053 words) is significant as a larger corpus means that there is a greater chance of repetitions in language, i.e. lexical terms and lexical lemmas, for a pattern to occur and be notable in the language used. Hence, the results of the analysis can be considered as more reliable due to the fact that a large corpus of natural language has been appraised. However, some studies have commented that there is more notable homogeneity in a smaller corpus (*see* Adolphs 2006 as cited in Bond *et al.*, 2018), which makes a smaller corpus more valid.

Several limitations can be identified. Firstly, the long-term effect(s) of TLT as an approach, regarding participants’ ability to sustain confidence and influence as leaders, cannot be fully determined from this evaluation study. Appraising leadership programmes is complex, in terms of individual influence, as the development process continues well beyond the learning experience. Second, while an increase in leadership capabilities at the individual level may be observable immediately after the programme, greater individual leadership growth and impact on the wider health-care system is likely to take place several years after completion of the leadership programme (*see* Brown *et al.*, 2022). Therefore, to achieve a comprehensive understanding of the effect of leadership programmes, it is crucial to implement validated measures that can assess both short and long-term outcomes. Moreover, as the evaluation shifts from short-term to long term-outcomes, so does the socio-ecological level of measurement which will progress from the individual to organisational, community and eventually towards measuring impact at a societal level. We aim to address these shortfalls by undertaking a longitudinal study, using robust measures of self-efficacy, and asking specific questions to programme alumni relating to the wider impact of these programmes on participants’ career status and quality improvements relating directly to patient care.

## Conclusion

Applying the principles of TLT to the development of health-care leaders has the potential to create socially and strategically confident leaders who are influential in creating positive changes across a variety of clinical environments. Future evaluation will explore in depth the personal transformational leadership journey over time, along with the implications of this on policy, practice and the promotion of clinical excellence.

## Figures and Tables

**Table 1.** First pattern of language observed in relation to the word-type confidence

the leadership journey has helped to develop my	**confidence**	and structure my leadership goals
me with a lot of opportunities to develop my	**confidence**	in leading, guiding and influencing
learning and courses enable me to gain more	**confidence**	in my leadership skill
I feel I have developed	**confidence**	and clarity in and on my own leadership style
This programme has enabled me to ooze with	**confidence**	and lead the team in a manner I did
I enjoyed the	**confidence**	building and transformation it instils as a leader
helped me gain more	**confidence**	in leading
I have gained greater	**confidence**	in my own ability as a system leader
better understanding of my leadership style, as a result my	**confidence**	in my abilities and skills has grown dramatically
in everything that I learnt in this course, my	**confidence**	as leader is improved
a boost in my	**confidence**	and self-awareness to become an effective healthcare leader
the course has enabled me to grow/develop my professional	**confidence**	by equipping me with leadership skills
This helped me a lot in gaining my	**confidence**	and gauging my potential as a future leader
(blinded) programme has helped me to build up my	**confidence**	as a leader
this is all just adding on to my	**confidence**	and shaping me into becoming a better leader
Overall, my	**confidence**	and leadership skills have noticeably improved
Wonderful experience, grown in	**confidence**	and leading others in service provision
I have further developed my	**confidence**	in my own ability to lead change
I have grown in	**confidence**	to become a good leader
my skills as a leader. As my	**confidence**	in this area has grown
gave me the	**confidence**	to step up and develop further in my leadership journey
(blinded) leadership program has provided me the	**confidence**	tools and drive to become an effective leader
has really enhanced my	**confidence**	as a leader
I have been given the skill set and increasing	**confidence**	to become a better leader
My	**confidence**	and abilities as a leader has improved during this
My self-	**confidence**	has really improved massively since the leadership course
leadership and my impact on others. I have gained	**confidence**	as a leader and have been shown ways to
I am very grateful. I have gained	**confidence**	in my leadership journey and now take active part
I now feel fairly	**confident**	with my style of leadership

**Table 2.** Second pattern of language observed in relation to the word-type influence

thinking much more about my potential to	**influence**	on a larger scale
shape my style to	**influence**	positive change
I am able to	**influence**	and guide the team towards a common goal
transformed my perspective of leadership, and how to	**influence**	change within an organisation
the experience I have gained from this program to	**influence**	positive change within my team
position as we are to	**influence**	systems for better patient care
I am able to lead and	**influence**	change
in the near future, so that I can lead and	**influence**	on a larger scale
starting to understand how to make an impact and	**influence**	change
made me more confident to speak out and	**influence**	improvements in patient care and health outcomes
I see many ways that I can	**influence**	change within The Florence Nightingale Foundation
dialogue on a strategic level with management to help	**influence**	change
(blinded) has helped me to further understand my	**influence**	in leading and driving change
I think harbouring this motivation, can only	**influence**	positive changes for the future
able to contribute to making a positive	**influence**	on my patients and colleagues

**Table 3.** Third pattern of language observed in relation to the notion of self-awareness

the programme started by triggering self-	**awareness**	understanding of where I was as a professional
In addition, a boost in my confidence and self-	**awareness**	to become effective and reliable healthcare provider
I have developed self-	**awareness**	and learned more about my personality type
The scholarship has helped increase self-	**awareness**	and identify areas for development
has given me time out to focus on self; self-	**awareness**	and self-development
They raised my self-	**awareness**	awareness of those around me and made me believe
highlighting positives and avenues for growth and	**awareness**	This course has taken me on a journey
The leadership programme has improved my own	**awareness**	of myself, my colleagues and others
A lot of self-learning and	**awareness**	has come as a result of this course
halfway through the program, and I have developed an	**awareness**	of my personal styles and approaches
a lot about myself my personality to which I had an	**awareness**	of however, this programme has highlighted
development; personal growth; increasing knowledge,	**awareness**	and information on leadership
perfect opportunity to gain a greater sense of	**awareness**	regarding my personal approach to leadership
My development has centred mainly of personal	**awareness**	of whom I am, how I work
focused on identifying my leadership style, my self	**awareness**	and how to develop my effectiveness

**Table 4.** Fourth pattern of language observed in relation to the word-type insight

I have gained an	**insight**	into how I can pursue leadership/managerial roles
I have also gained	**insight**	into using resources and sharing my vision
I have gained an	**insight**	into more strategic thinking
Not what I expected but gave me so much	**insight**	into myself. Giving renewed passion for my role
it has given me some good	**insight**	to leadership, and skills to take forward in my
has enabled me to get an	**insight**	into the different stakeholder requirements
It gave me a really good	**insight**	into the different ways people think and interact
I have gained so much	**insight**	into myself and my leadership style
has given me the	**insight**	into the roles and responsibilities of senior leadership
has given me	**insight**	into how to implement positive changes
I have gained a fair amount of knowledge and	**insight**	into leadership and quality improvement.
The course has offered me great	**insight**	into myself and how I can impact

**Table 5.** Fifth pattern of language observed in relation to the word-type insight

questioning how can I improve myself to have the	**impact**	I want to have in others
It’s made me realise the	**impact**	I have on others around me, small and big
It made me realise the	**impact**	I have to others, the way I speak
I have learnt so much about myself and the	**impact**	that people do have.
vital component is understanding self and the	**impact**	on others, as well as effect role modelling
I am starting to understand how to make an	**impact**	and influence change
see the bigger picture and consider my	**impact**	and influence in my current role
enhanced my knowledge of leadership and my	**impact**	on others
reflect and learn about myself, my style, and my	**impact**	on those around me
great insight into myself and how I can	**impact.**	I feel that this has given me a drive
learn very quickly how my style can	**impact**	and influence other people that I am responsible for
about myself and this has had a huge	**impact**	on my career already
I have been more mindful of myself, how I	**impact**	others and myself by how I lead at work
I have learnt how I present myself can have an	**impact**	on how I am perceived

**Table 6.** Final pattern of language observed in relation to the word-type impact

I have learnt that I can have a positive	**impact**	at work and be a leader
where possible and feel I have a positive	**impact**	on the ward I work on
thereby enabling me to have a positive	**impact**	in all the projects that I take on
motivate people to make a positive	**impact**	to patient care and to the well-being of
is going to have a positive	**impact**	in my career. Very thankful
believe can have a positive	**impact**	in advocating positive change. I feel more energised
things that I can change and make a positive	**impact**	to be open and engage in difficult conversations
mine and make a positive	**impact**	I feel my communication is now focused
to learn ideas from others to make a positive	**impact**	I was genuinely delighted to take part in this
access better and make a more positive	**impact**	in future situations and scenarios.
which had a significant positive	**impact**	on my leadership development
create the positive	**impact**	giving the best possible outcome to patient care
how to implement changes and create positive	**impact**	for the patients and for The Florence Nightingale Foundation
It really had a positive	**impact**	on my professional and personal life
thus, having a positive	**impact**	and outcome to patient care
I have made a positive	**impact**	in my job through showing strong leadership
I’ve been making a positive	**impact.**	This has been the best course I have ever
challenging but rewarding to see the positive	**impact**	the project had on the ward where I work

## Notes

1. The concept of transformational leadership was introduced by Burns in 1978 and is defined as a process whereby “leaders and followers help each other to advance to a higher level of morale and motivation”. Ideally, the end goal is to develop followers into leaders.

2. Agenda for Change (AfC) is the current National Health Service (NHS) grading and pay system for NHS staff, except for doctors, dentists, apprentices and some senior managers. Whilst not universally used by all participants, e.g. participants from the Army, it represents the career pay grading scale used by most participants and was used in the questionnaire for this reason. Where a programme participant used a different pay system scale, they were asked to submit this so it could be matched to the AfC equivalent.
